# Pre-treatment serum phosphate as a metabolic correlate of live birth after IVF/ICSI: a retrospective cohort study

**DOI:** 10.3389/fendo.2026.1938890

**Published:** 2026-08-27

**Authors:** Zhilong Wang, Xiaoyue Shen, Qingqing Shi, Yuan Yan, Chuanming Liu, Ruiwei Jiang, Feifei Lu, Lingjuan Wang, Jie Mei, Yue Jiang, Weihong Zhou, Na Kong

**Affiliations:** 1 Center for Reproductive Medicine and Obstetrics and Gynecology, Nanjing Drum Tower Hospital, Affiliated Hospital of Medical School, Nanjing University, Nanjing, China; 2 Center for Molecular Reproductive Medicine, Nanjing University, Nanjing, China; 3 Department of Health Management Center, Nanjing Drum Tower Hospital, Affiliated Hospital of Medical School, Nanjing University, Nanjing, China

**Keywords:** folliculogenesis, ICSI, IVF, live birth, metabolic biomarker, oocyte competence, phosphate homeostasis, serum phosphate

## Abstract

**Background:**

Oocyte developmental competence is influenced by the systemic metabolic milieu during folliculogenesis. Inorganic phosphate is essential for ATP synthesis, nucleic acid metabolism, and phosphorylation-dependent signalling, but its relationship with assisted reproductive technology outcomes remains unclear. We investigated whether pre-treatment fasting serum phosphate was associated with live birth after first IVF/ICSI fresh embryo transfer.

**Methods:**

This retrospective cohort study included 2, 226 women aged 20–42 years undergoing their first IVF/ICSI cycle with fresh embryo transfer between January 2022 and June 2024. Serum phosphate was measured before ovarian stimulation and analysed as quartiles and per 1-SD increase (0.146 mmol/L). Multivariable logistic regression assessed associations with live birth, adjusting for demographic, ovarian reserve, metabolic, and treatment-related factors. Restricted cubic splines evaluated dose–response relationships. Measurement-window analyses stratified participants by the interval between biochemistry assessment and oocyte retrieval.

**Results:**

The overall live birth rate was 60%, increasing from 56% in the lowest phosphate quartile to 66% in the highest (P for trend = 0.003). Each 1-SD increase in serum phosphate was associated with higher odds of live birth (adjusted OR 1.15, 95% CI 1.06–1.26; P = 0.002), with a linear dose–response (P for non-linearity = 0.305). Serum phosphate was not associated with MII oocyte count or usable embryo count. In measurement-window analyses, the association was consistent in the 60–120 day window (n = 1, 485; adjusted OR 1.18, 95% CI 1.03–1.35) and the 120–180 day window (n = 637; adjusted OR 1.16, 95% CI 1.01–1.34). The 0–60 day stratum was underpowered (n = 104). Additional adjustment for calcium, alkaline phosphatase, fasting glucose, and trigger-day progesterone did not materially alter the results.

**Conclusions:**

Higher pre-treatment serum phosphate was associated with higher odds of live birth after IVF/ICSI, independent of oocyte yield. External validation and mechanistic studies are required before clinical application.

## Introduction

The success of *in vitro* fertilisation (IVF) depends fundamentally on oocyte developmental competence, defined as the gamete’s capacity to support fertilization, embryonic development, implantation, and ultimately live birth (1, 2). The acquisition of this developmental competence occurs over the months-long course of folliculogenesis; consequently, the systemic metabolic milieu during this critical window is increasingly recognised as a determinant of reproductive outcomes (2, 3).

Research on the metabolic regulation of fertility has focused predominantly on macronutrients, lipid metabolism, and endocrine disruptors (3–8). By contrast, essential inorganic minerals have received far less attention. Inorganic phosphate is foundational to cellular bioenergetics and signalling: it is a constituent of adenosine triphosphate (ATP), nucleic acid metabolism, and membrane phospholipids, and underpins signal transduction through reversible protein phosphorylation (9, 10). These processes are essential for energy-demanding events during oocyte maturation, including meiotic spindle assembly, cytoskeletal remodelling, and chromosomal segregation (11, 12). Serum phosphate is regulated by a complex endocrine and metabolic network involving parathyroid hormone (PTH), vitamin D, fibroblast growth factor 23 (FGF23), klotho, renal phosphate handling, bone metabolism, and dietary intake (13–15). Accordingly, fasting serum phosphate may reflect the integrated state of several metabolic pathways rather than a direct causal exposure. Whether this systemic marker is associated with ART outcomes remains uncertain.

Evidence linking circulating phosphate to reproductive outcomes remain sparse. Whilst dietary phosphate intake appears unrelated to ovulatory infertility (16), dietary patterns rich in phosphate-containing foods—particularly dairy products—have been associated with improved fecundability in preconception cohort studies (17). In the only clinical assisted reproductive technology (ART) study to date, Wulff et al. reported that follicular fluid phosphate exceeds serum concentrations and that both compartments correlate significantly; however, they detected no association with live birth in a cohort of 110 patients (18). These inconsistencies likely reflect limited statistical power and the inherent inability of natural fertility studies to disentangle ovarian response (oocyte yield) from oocyte developmental competence.

The IVF setting allows ovarian response, oocyte maturation, fertilization, embryo development, and live birth to be evaluated within the same treatment cycle. We hypothesised that higher pre-treatment fasting serum phosphate would be positively associated with live birth, reflecting a role in oocyte developmental competence independent of ovarian response. We also evaluated oocyte and embryo laboratory outcomes to determine whether any association was reflected in oocyte maturation, fertilization, or embryo utilization.

## Materials and methods

### Study design and population

This retrospective cohort study was conducted at the Center for Reproductive Medicine and Obstetrics and Gynecology, Nanjing Drum Tower Hospital, Affiliated Hospital of Medical School, Nanjing University, and included women aged 20–42 years undergoing their first IVF or ICSI cycle with fresh embryo transfer between January 2022 and June 2024. Data were extracted from linked electronic medical records and embryology databases. The study was reported according to the Strengthening the Reporting of Observational Studies in Epidemiology (STROBE) guidelines.

**Figure 1:**
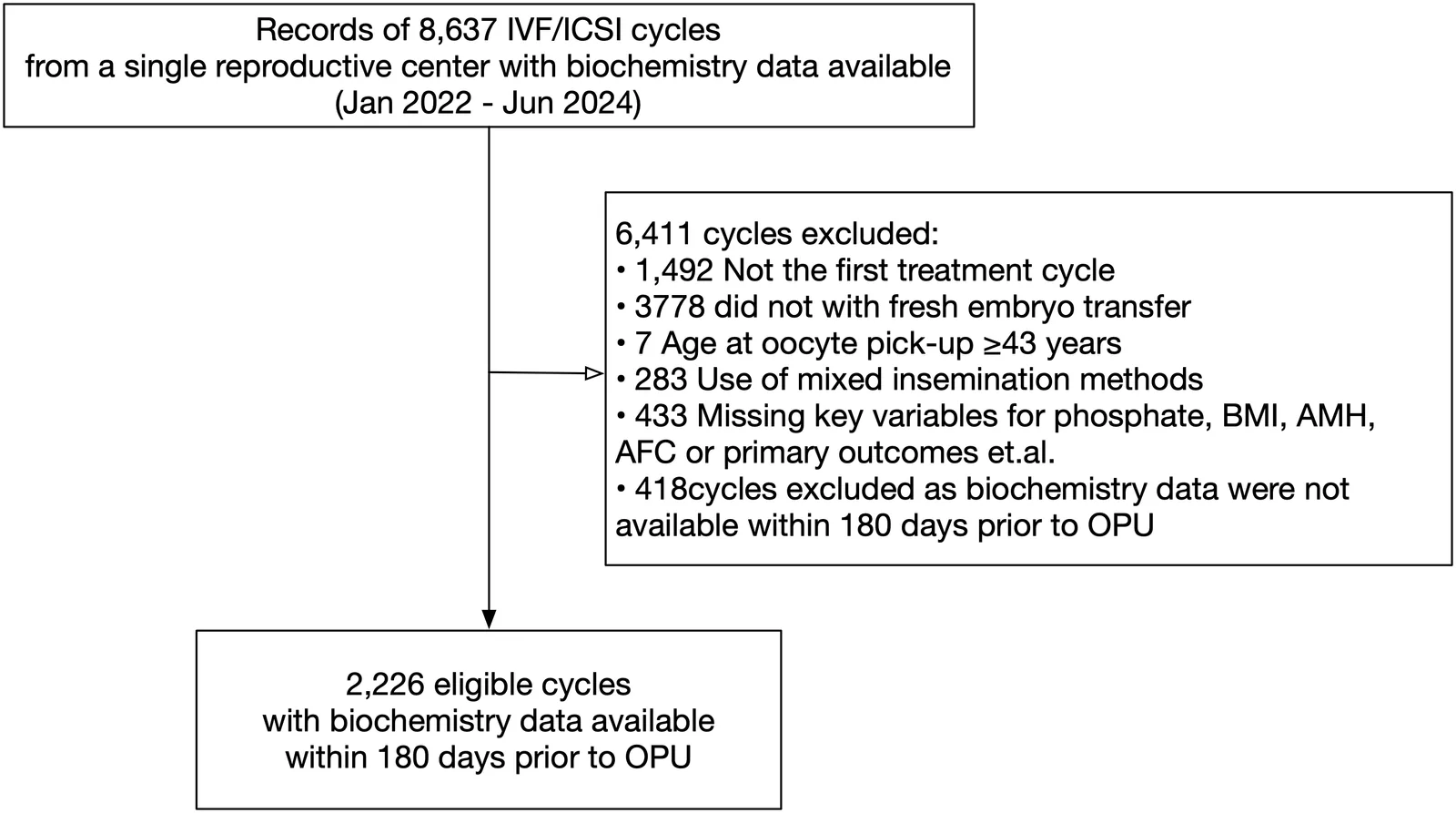
Study flow chart. Flow diagram showing participant selection. Of 4, 852 fresh IVF/ICSI embryo transfer cycles with available biochemistry data between January 2022 and June 2024, 2, 226 women met eligibility criteria and were included in the primary analysis. Numbers excluded and reasons for exclusion are shown. IVF, *in vitro* fertilization; ICSI, intracytoplasmic sperm injection; OPU, oocyte pick-up; BMI, body mass index; AMH, anti-Müllerian hormone; AFC, antral follicle count.

Eligibility criteria required a documented first oocyte retrieval cycle with at least one fresh embryo transfer. Exclusion criteria were applied sequentially: (i) non-first IVF/ICSI cycle; (ii) no fresh embryo transfer performed; (iii) mixed insemination (simultaneous IVF and ICSI); (iv) missing key covariates (BMI, AMH, AFC, infertility type, or infertility duration); and (v) biochemistry-to-retrieval interval >180 days. This 180-day window was selected to overlap with the approximately 85-day duration of human antral follicle development (19), whilst allowing for practical clinical scheduling. The number excluded at each step is shown in **Figure 1**.

### Exposure assessment

Fasting serum phosphate (mmol/L) was measured in venous blood samples collected after a minimum 8-hour overnight fast using a colorimetric assay (phosphomolybdate method, Mindray BS2800 automated analyser). Calcium, alkaline phosphatase (ALP), and fasting glucose were measured on the same platform. AMH and estradiol were measured on the Roche Cobas C8000. Estimated glomerular filtration rate (eGFR) was calculated using the MDRD equation. Serum phosphate was analysed as quartiles (Q1–Q4) and continuously as per 1-SD increase (0.146 mmol/L). The interval between biochemistry assessment and oocyte retrieval was categorised as 0–60, 60–120, and 120–180 days. The 60–120 day window was considered biologically relevant because it plausibly overlaps with a substantial portion of antral follicle development, when oocyte–granulosa cell metabolic coupling and acquisition of developmental competence are established (19).

### Outcomes

The primary outcome was live birth, defined as delivery of at least one live infant at ≥28 weeks of gestation, ascertained from hospital delivery records and postpartum follow-up documented in the electronic medical record. Secondary outcomes included clinical pregnancy, singleton live birth, healthy baby (singleton live birth without preterm birth or birth defects), and miscarriage. Laboratory outcomes were MII oocyte count and usable embryo count, classified per center criteria (20). Specifically, cleavage-stage embryos were considered usable if they met Scott’s criteria (Grade I or II with ≥6 cells). Blastocysts (Day 5 or 6) were graded using the Gardner criteria; those with an expansion grade ≥3 and inner cell mass/trophectoderm quality of A, B, or C (excluding CC grade) were defined as usable. Additional embryological indicators were reported descriptively. The oocyte maturation index was calculated as: total MII oocytes/total oocytes retrieved. The aggregate fertilization rate was calculated as: total 2PN embryos/total MII oocytes. The aggregate embryo utilization rate was calculated as: total usable embryos/total 2PN embryos. This definition follows the Vienna consensus on ART laboratory performance indicators, which defines embryo utilization as the number of embryos suitable for transfer or cryopreservation relative to the number of normally fertilized 2PN oocytes (21).

### Covariates

The following variables were identified *a priori* as potential confounders based on clinical knowledge and directed acyclic graph reasoning: maternal age at oocyte retrieval (years), BMI (kg/m²), AMH, AFC (total count on baseline transvaginal ultrasound), eGFR (mL/min/1.73 m²), infertility type (primary vs. secondary), infertility duration (years), fertilisation method (conventional IVF vs. ICSI), peak oestradiol on the day of trigger administration, endometrial thickness on the day of trigger administration (mm), and number and developmental stage (cleavage vs. blastocyst) of embryos transferred.

### Statistical analysis

Baseline characteristics were summarised by phosphate quartile as mean ± standard deviation (SD) or median (interquartile range [IQR]) for continuous variables and number (percentage) for categorical variables. Between-group differences were assessed using the Kruskal–Wallis test for continuous variables and the Pearson’s chi-squared test or Fisher’s exact test for categorical variables.

Multivariable logistic regression was used for binary outcomes, and negative binomial regression was used for count outcomes given evidence of overdispersion. Three nested models were prespecified: Model 1, unadjusted; Model 2, adjusted for age, BMI, AMH, AFC, and eGFR; and Model 3, additionally adjusted for infertility type, infertility duration, fertilisation method, peak oestradiol, endometrial thickness, and number and developmental stage of embryos transferred. For MII oocyte count, Model 3 included Model 2 covariates plus infertility type and infertility duration only to avoid overadjustment by treatment-related variables; for usable embryo count, fertilisation method was additionally included.

Restricted cubic splines with four knots placed at the 5th, 35th, 65th, and 95th percentiles of the phosphate distribution were used to characterize the dose–response relationship between serum phosphate and live birth. The median phosphate concentration was used as the reference value.

Measurement-window analyses evaluated associations within the 0–60, 60–120, and 120–180 day strata. Interaction testing used multiplicative product terms (phosphate × measurement window) in fully adjusted models. Additional exploratory subgroup analyses assessed potential effect modification by maternal age (≤35 vs. >35 years), BMI (<24.0 vs. ≥24.0 kg/m², consistent with Chinese adult classification), and fertilisation method (conventional IVF vs. ICSI).

Sensitivity analyses included additional adjustment for trigger-day progesterone to account for premature luteinization, and for serum calcium, ALP, and fasting glucose (individually and simultaneously) to evaluate whether the phosphate–live birth association was independent of the PTH–calcium axis, bone turnover pathways, and insulin-mediated phosphate metabolism.

Two-sided P < 0.05 was considered statistically significant. Subgroup and interaction analyses were considered exploratory. Analyses were performed using R version 4.5 (R Foundation for Statistical Computing, Vienna, Austria).

## Results

### Study population

Between January 2022 and June 2024, 4, 852 fresh IVF/ICSI embryo transfer cycles with available local biochemistry data were identified. After sequential application of exclusion criteria, 2, 226 women were included in the analytic cohort (**Figure 1**). The median interval between biochemistry assessment and oocyte retrieval was 98 days (IQR 80–125 days). Among included women, 104 had phosphate measured 0–60 days before retrieval, 1, 485 at 60–120 days, and 637 at 120–180 days. The mean age was 31.1 ± 3.9 years, mean BMI was 23.5 ± 3.5 kg/m², mean AFC was 19.3 ± 7.9, and mean serum phosphate was 1.17 ± 0.15 mmol/L. Phosphate quartile boundaries were Q1 ≤1.07 mmol/L, Q2 1.08–1.17 mmol/L, Q3 1.18–1.27 mmol/L, and Q4 ≥1.28 mmol/L. Across quartiles, women in Q1 were slightly older and had higher BMI and lower AFC than those in Q4 (**Table 1**).

**Table 1:** Baseline demographic, clinical, and laboratory characteristics of the study cohort stratified by pre-treatment serum phosphate quartile (N = 2, 226).

Serum phosphorus quartiles						
Characteristic	Overall N = 2, 226	Q1 N = 591	Q2 N = 558	Q3 N = 542	Q4 N = 535	p-value
Age (years), Mean (SD)	31 (4)	32 (4)	31 (4)	31 (4)	31 (4)	<0.001
BMI (kg/m²), Mean (SD)	23.5 (3.5)	23.8 (3.5)	23.5 (3.5)	23.2 (3.5)	23.5 (3.7)	0.024
AMH (ng/mL), Mean (SD)	3.2 (1.8)	3.1 (1.8)	3.2 (1.9)	3.2 (1.8)	3.4 (1.9)	0.26
Antral follicle count, Mean (SD)	19 (8)	19 (8)	19 (8)	19 (8)	20 (8)	0.022
Infertility type, n (%)						0.61
Primary	1, 150 (52%)	297 (50%)	287 (51%)	293 (54%)	273 (51%)	
Secondary	1, 076 (48%)	294 (50%)	271 (49%)	249 (46%)	262 (49%)	
Infertility duration (years), Mean (SD)	3 (2)	3 (2)	3 (2)	3 (2)	3 (2)	0.78
eGFR, Mean (SD)	132 (22)	133 (22)	132 (23)	132 (22)	130 (23)	0.044
Serum phosphorus (mmol/L), Mean (SD)	1.17 (0.15)	1.00 (0.07)	1.12 (0.03)	1.21 (0.03)	1.36 (0.09)	<0.001
Stimulation duration (days), Mean (SD)	12 (2)	12 (2)	12 (2)	12 (3)	12 (3)	0.38
Total gonadotropin dose (IU), Mean (SD)	2, 094 (712)	2, 072 (724)	2, 092 (682)	2, 104 (689)	2, 112 (751)	0.66
Trigger day progesterone (ng/mL), Mean (SD)	0.71 (0.37)	0.71 (0.35)	0.72 (0.41)	0.72 (0.37)	0.70 (0.35)	0.72
Serum E2 peak value(pg/mL), Mean (SD)	2, 091 (1, 160)	2, 105 (1, 215)	2, 145 (1, 168)	2, 038 (1, 068)	2, 072 (1, 180)	0.5
Oocytes collected, Mean (SD)	10 (4)	10 (4)	10 (4)	10 (4)	10 (4)	0.75
MII oocytes, Mean (SD)	9 (4)	9 (4)	9 (4)	9 (3)	9 (4)	0.73
Ovarian Sensitivity Index (OSI), Mean (SD)	5.35 (2.95)	5.41 (3.00)	5.38 (2.98)	5.26 (2.96)	5.36 (2.84)	0.79
Fertilization method, n (%)						0.068
ICSI	389 (17%)	124 (21%)	94 (17%)	85 (16%)	86 (16%)	
IVF	1, 837 (83%)	467 (79%)	464 (83%)	457 (84%)	449 (84%)	
2PN embryo count, Mean (SD)	7 (3)	7 (3)	8 (3)	7 (3)	8 (3)	0.64
Usable embryos, Mean (SD)	4 (2)	4 (2)	4 (2)	4 (2)	4 (2)	0.87
Transferred embryo count, n (%)						0.71
1	1, 773 (80%)	472 (80%)	438 (78%)	440 (81%)	423 (79%)	
2	453 (20%)	119 (20%)	120 (22%)	102 (19%)	112 (21%)	
Embryo stage, n (%)						0.34
Blastocyst	1, 274 (57%)	322 (54%)	316 (57%)	319 (59%)	317 (59%)	
Cleavage	952 (43%)	269 (46%)	242 (43%)	223 (41%)	218 (41%)	
Endometrium thickness(mm), Mean (SD)	12.2 (2.6)	12.2 (2.6)	12.0 (2.7)	12.4 (2.4)	12.3 (2.6)	0.24
Clinical Pregnancy, n (%)	1, 525 (69%)	386 (65%)	378 (68%)	369 (68%)	392 (73%)	0.035
Miscarriage, n (%)	168 (7.5%)	51 (8.6%)	38 (6.8%)	47 (8.7%)	32 (6.0%)	0.23
Live Birth, n (%)	1, 330 (60%)	329 (56%)	329 (59%)	317 (58%)	355 (66%)	0.003
Singleton Live Birth, n (%)	1, 197 (54%)	289 (49%)	307 (55%)	281 (52%)	320 (60%)	0.002
Healthy baby rate, n (%)	1, 069 (48%)	259 (44%)	270 (48%)	254 (47%)	286 (53%)	0.013

Data are presented as mean (SD) for continuous variables and n (%) for categorical variables. P values were calculated using Kruskal-Wallis rank sum test for continuous variables and Pearson’s Chi-squared test for categorical variables. AMH, anti-Müllerian hormone; BMI, body mass index; eGFR, estimated glomerular filtration rate; ICSI, intracytoplasmic sperm injection; IVF, *in vitro* fertilization;MII, metaphase II.

### Primary analysis

**Figure 2:**
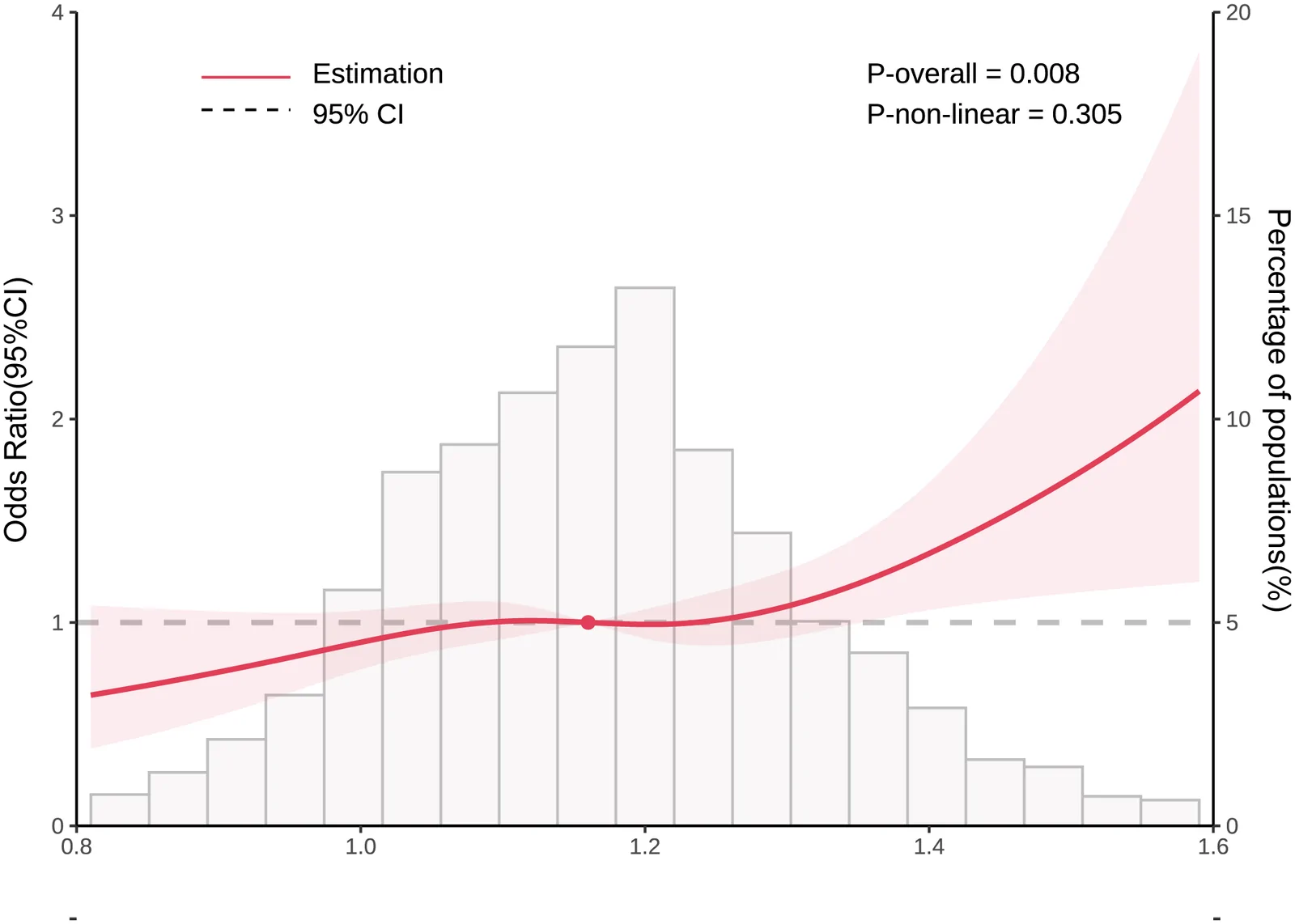
Dose–response relationship between serum phosphate and live birth. Adjusted odds ratio (OR) for live birth as a function of serum phosphate (mmol/L), modelled using restricted cubic splines with four knots. The shaded area represents the 95% confidence interval. The reference value was set at the median phosphate concentration (1.17 mmol/L). The model was adjusted for age, BMI, AMH, AFC, eGFR, infertility characteristics, fertilization method, endometrial thickness, and embryo-transfer characteristics. The histogram shows the distribution of serum phosphate.

The overall live birth rate was 60%. Live birth rates increased monotonically from 56% (Q1) to 66% (Q4) (P for trend = 0.003). Restricted cubic spline analysis confirmed a linear relationship between serum phosphate and the odds of live birth across the observed concentration range (P for overall association = 0.008; P for non-linearity = 0.305) (**Figure 2**).

In fully adjusted models, Q4 compared with Q1 yielded aOR 1.40 (95% CI 1.09–1.80; P = 0.008); per 1-SD increase, aOR 1.15 (1.06–1.26; P = 0.002). Results from Models 1 and 2 were directionally consistent and of similar magnitude, indicating limited confounding by the included covariates (**Table 2**).

**Table 2:** Association between pre-treatment serum phosphate and clinical pregnancy outcomes: sequential multivariable logistic regression models.

		Model 1		Model 2		Model 3	
Outcomes	Characteristic	OR (95% CI)	p-value	OR (95% CI)	p-value	OR (95% CI)	p-value
Live Birth	Phosphate (per 1-SD)	1.21 (1.11 to 1.32)	<0.001	1.17 (1.07 to 1.28)	<0.001	1.15 (1.06 to 1.26)	0.002
	Q1	—		—		—	
	Q2	1.14 (0.91 to 1.45)	0.26	1.11 (0.87 to 1.40)	0.4	1.08 (0.85 to 1.38)	0.51
	Q3	1.12 (0.89 to 1.42)	0.34	1.05 (0.82 to 1.33)	0.72	1.02 (0.80 to 1.30)	0.87
	Q4	1.57 (1.23 to 2.00)	<0.001	1.44 (1.13 to 1.85)	0.003	1.40 (1.09 to 1.80)	0.008
Singleton Live Birth	Phosphate (per 1-SD)	1.18 (1.09 to 1.29)	<0.001	1.16 (1.06 to 1.26)	<0.001	1.16 (1.06 to 1.27)	<0.001
	Q1	—		—		—	
	Q2	1.28 (1.01 to 1.61)	0.038	1.25 (0.99 to 1.58)	0.06	1.26 (0.99 to 1.60)	0.058
	Q3	1.13 (0.89 to 1.42)	0.32	1.08 (0.85 to 1.37)	0.54	1.05 (0.83 to 1.34)	0.66
	Q4	1.56 (1.23 to 1.97)	<0.001	1.47 (1.15 to 1.87)	0.002	1.46 (1.14 to 1.87)	0.002
Healthy Baby	Phosphate (per 1-SD)	1.17 (1.08 to 1.27)	<0.001	1.14 (1.04 to 1.24)	0.003	1.14 (1.04 to 1.24)	0.004
	Q1	—		—		—	
	Q2	1.20 (0.95 to 1.52)	0.12	1.16 (0.92 to 1.47)	0.21	1.17 (0.92 to 1.48)	0.2
	Q3	1.13 (0.89 to 1.43)	0.3	1.06 (0.83 to 1.34)	0.65	1.04 (0.82 to 1.32)	0.77
	Q4	1.47 (1.16 to 1.86)	0.001	1.37 (1.08 to 1.74)	0.01	1.36 (1.07 to 1.73)	0.012
Clinical Pregnancy	Phosphate (per 1-SD)	1.17 (1.07 to 1.28)	<0.001	1.14 (1.04 to 1.25)	0.005	1.13 (1.02 to 1.24)	0.014
	Q1	—		—		—	
	Q2	1.12 (0.87 to 1.43)	0.38	1.09 (0.85 to 1.40)	0.5	1.07 (0.83 to 1.37)	0.61
	Q3	1.13 (0.88 to 1.45)	0.32	1.08 (0.84 to 1.40)	0.53	1.06 (0.82 to 1.36)	0.67
	Q4	1.46 (1.13 to 1.88)	0.004	1.36 (1.05 to 1.76)	0.022	1.31 (1.01 to 1.70)	0.046
Miscarriage	Phosphate (per 1-SD)	0.89 (0.76 to 1.04)	0.15	0.92 (0.78 to 1.08)	0.32	0.92 (0.78 to 1.08)	0.32
	Q1	—		—		—	
	Q2	0.77 (0.50 to 1.19)	0.25	0.80 (0.51 to 1.24)	0.32	0.81 (0.52 to 1.26)	0.35
	Q3	1.01 (0.66 to 1.52)	0.98	1.12 (0.73 to 1.70)	0.61	1.11 (0.73 to 1.70)	0.62
	Q4	0.67 (0.42 to 1.06)	0.091	0.73 (0.45 to 1.15)	0.18	0.73 (0.45 to 1.15)	0.18

Data are presented as odds ratios (OR) with 95% confidence intervals (CI). Phosphate was modeled as quartiles (reference: Q1, ≤1.07 mmol/L), and per 1-SD increase (0.146 mmol/L). Model 1: unadjusted. Model 2: adjusted for maternal age, body mass index, anti-Müllerian hormone, antral follicle count, and estimated glomerular filtration rate. Model 3: Model 2 plus infertility type, infertility duration, fertilization method, peak estradiol, endometrial thickness, number and developmental stage of embryos transferred. CI, Confidence Interval; OR, Odds Ratio.

Secondary clinical outcomes were directionally consistent with the primary outcome. Per 1-SD increase in serum phosphate, the adjusted OR was 1.16 for singleton live birth (95% CI 1.06–1.27; P < 0.001) and 1.13 for clinical pregnancy (95% CI 1.02–1.24; P = 0.014). Serum phosphate was not significantly associated with miscarriage (aOR 0.92, 95% CI 0.78–1.08; P = 0.32) (**Table 2**).

### Associations with oocyte yield and embryo outcomes

Serum phosphate was not associated with MII oocyte count (adjusted incidence rate ratio [aIRR] 0.99, 95% CI 0.97–1.00; P = 0.17) or usable embryo count (aIRR 0.99, 95% CI 0.96–1.01; P = 0.18) (**Table 3**). A total of 21, 934 oocytes were retrieved, including 19, 472 MII oocytes. The aggregate oocyte maturation index was 0.89 and remained similar across phosphate quartiles. The aggregate embryo utilization rate was 59.7% overall, ranging from 58.8% to 60.5% across phosphate quartiles (**Supplementary Table 2**). The dissociation between the positive association with live birth and the absence of association with oocyte or embryo quantity supports an interpretation related to embryo developmental competence rather than ovarian response.

**Table 3:** Association between pre-treatment serum phosphate and laboratory outcomes: sequential multivariable negative binomial regression models.

		Model 1		Model 2		Model 3	
Outcomes	Characteristic	IRR (95% CI)	p-value	IRR (95% CI)	p-value	IRR (95% CI)	p-value
MII Oocyte Count	Phosphate (per 1-SD)	1.00 (0.99 to 1.02)	0.6	0.99 (0.97 to 1.00)	0.18	0.99 (0.97 to 1.00)	0.17
	Q1	—		—		—	
	Q2	1.02 (0.97 to 1.07)	0.38	1.00 (0.96 to 1.05)	0.86	1.00 (0.96 to 1.05)	0.87
	Q3	1.00 (0.95 to 1.05)	0.96	0.98 (0.94 to 1.03)	0.39	0.98 (0.94 to 1.03)	0.4
	Q4	1.03 (0.98 to 1.08)	0.26	0.99 (0.94 to 1.03)	0.58	0.99 (0.94 to 1.03)	0.54
Usable Embryo Count	Phosphate (per 1-SD)	1.00 (0.98 to 1.02)	0.9	0.99 (0.97 to 1.01)	0.23	0.99 (0.96 to 1.01)	0.18
	Q1	—		—		—	
	Q2	1.02 (0.96 to 1.08)	0.6	1.00 (0.94 to 1.06)	0.99	1.00 (0.94 to 1.06)	0.9
	Q3	0.98 (0.92 to 1.04)	0.48	0.96 (0.90 to 1.02)	0.15	0.95 (0.90 to 1.01)	0.12
	Q4	1.02 (0.95 to 1.08)	0.63	0.98 (0.92 to 1.04)	0.47	0.97 (0.92 to 1.03)	0.38

Data are presented as incidence rate ratios (IRR) with 95% confidence intervals (CI). Phosphate was modeled as quartiles (reference: Q1), and per 1-SD increase. Model 1: unadjusted. Model 2: adjusted for maternal age, body mass index, anti-Müllerian hormone, antral follicle count, and estimated glomerular filtration rate. Model 3 for MII oocyte count: Model 2 plus infertility type and infertility duration. Model 3 for usable embryo count: Model 2 plus infertility type, infertility duration, and fertilization method. MII, metaphase II; IRR, incidence rate ratio.

### Measurement-window analysis

**Figure 3:**
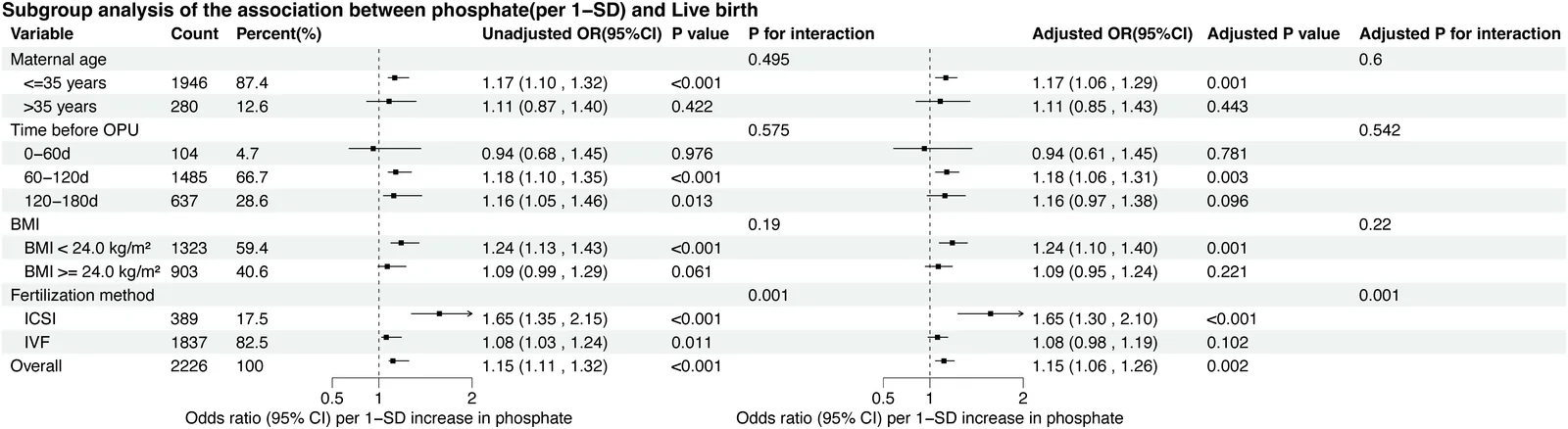
Subgroup analyses of the phosphate–live birth association. Forest plot showing adjusted odds ratios (aORs) and 95% confidence intervals (CIs) for live birth per 1-SD increase in serum phosphate (0.146 mmol/L) across prespecified subgroups. Left panel: unadjusted estimates; right panel: fully adjusted estimates. Subgroups were defined by age, measurement window, BMI, and fertilization method. The diamond represents the overall estimate, and the vertical dashed line indicates OR = 1.0. P values for interaction were derived from multiplicative interaction terms in fully adjusted models. Square size is proportional to subgroup sample size. IVF, *in vitro* fertilization; ICSI, intracytoplasmic sperm injection; BMI, body mass index; SD, standard deviation.

In measurement-window analyses, the phosphate–live birth association was observed in the core 60–120 day window, which included most participants (n = 1, 485). In this stratum, each 1-SD increase in serum phosphate was associated with higher odds of live birth (aOR 1.18, 95% CI 1.03–1.35). A similar association was observed in the 120–180 day window (n = 637; aOR 1.16, 95% CI 1.01–1.34). The 0–60 day stratum was small (n = 104) and showed a directionally similar but non-significant estimate (aOR 1.12, 95% CI 0.89–1.41; P = 0.33) (**Figure 3A**). *Post-hoc* calculation indicated that this stratum had approximately 10% power to detect the observed effect size. The phosphate × measurement-window interaction was not statistically significant (P for interaction = 0.30), supporting consistency of the association across the pre-treatment measurement windows, particularly within the 60–180 day period.

### Exploratory subgroup analyses

No significant effect modification was observed by maternal age (≤35 vs. >35 years; P for interaction = 0.60) or BMI (<24.0 vs. ≥24.0 kg/m²; P for interaction = 0.22). Exploratory analyses suggested possible heterogeneity by fertilization method, with a stronger association in ICSI cycles (aOR 1.65, 95% CI 1.30–2.10) than in conventional IVF cycles (aOR 1.08, 95% CI 0.98–1.19; P for interaction < 0.001). Because detailed male-factor characteristics were unavailable, this finding was considered exploratory and requires independent validation (**Figure 3**).

### Sensitivity analyses

The phosphate–live birth association was materially unchanged after additional adjustment for serum calcium, ALP, and fasting glucose(individually or simultaneously), and for trigger-day progesterone (aOR range 1.15–1.16; all P ≤ 0.002) (**Supplementary Table 1**). These findings suggest that the association was independent of the PTH–calcium axis, bone turnover, insulin-mediated phosphate metabolism, and premature luteinization.

## Discussion

In this retrospective cohort of 2, 226 women undergoing their first IVF/ICSI fresh embryo transfer cycle, higher pre-treatment fasting serum phosphate was associated with higher odds of live birth. The study population exhibited highly favorable baseline clinical and laboratory performance indicators: the overall live birth rate was 60%, the aggregate fertilization rate was 85%, the aggregate embryo utilization rate was 60%, and 87% of cycles achieved extra embryos cryopreservation. These key performance indicators (KPIs) align with expected international benchmarks for optimal ART laboratory performance (21). The association was linear, robust to adjustment for clinical and metabolic covariates, and independent of MII oocyte count and usable embryo count.

The main finding of this study is that pre-treatment serum phosphate was associated with reproductive outcomes but was unrelated to oocyte or embryo quantity. This dissociation suggests that phosphate may not simply a marker of ovarian response, oocyte maturation, or early embryo morphology. Instead, it may reflect a broader systemic metabolic milieu related to reproductive competence. This interpretation is biologically plausible but should remain cautious. Phosphate is required for ATP synthesis, nucleic acid metabolism, membrane phospholipid structure, and phosphorylation-dependent signalling cascades (9, 10). These processes are relevant to mitochondrial function, meiotic progression, chromosomal segregation, and early embryonic development (11, 12). However, serum phosphate is regulated by PTH, vitamin D, FGF23, klotho, renal phosphate handling, bone metabolism, and dietary intake (13–15). Because these regulators were not measured, we cannot determine whether the observed association reflects phosphate availability itself or related endocrine-metabolic pathways.

Prior evidence linking phosphate to ART outcomes is limited. Wulff et al. reported that follicular fluid phosphate concentrations were higher than paired serum concentrations and that serum and follicular fluid phosphate were correlated, suggesting potential systemic–intrafollicular coupling (18). However, their study did not detect an association with live birth, likely because the sample size was small. Our larger cohort provides greater statistical power and supports a modest but consistent association between serum phosphate and live birth, although external validation is required. Crucially, the independence of this association from calcium, alkaline phosphatase, and fasting glucose reduces the likelihood that our findings are merely secondary to major bone turnover or insulin-mediated mineral shifts.

The measurement-window analysis provides key biological and clinical insights. Human folliculogenesis from early follicle growth to ovulation spans several months, with antral follicle development occurring over approximately 85 days (19). Our pre-treatment measurement window (median 98 days before retrieval) thus captures the systemic metabolic state during the critical early stages of antral follicle growth. The association was observed in the 60–120 day window and remained consistent in the 120–180 day window, whereas the 0–60 day stratum was underpowered. This pattern is compatible with the hypothesis that fasting serum phosphate reflects a relatively stable pre-treatment metabolic state during follicular development rather than an acute peri-retrieval exposure. Measuring phosphate before ovarian stimulation also avoids potential confounding by supraphysiological estradiol levels during stimulation.

From a clinical perspective, fasting serum phosphate is an inexpensive, universally available marker. However, the present findings do not support empirical phosphate supplementation or clinical decision-making based on phosphate levels. Chronic phosphate excess carries well-established cardiovascular and renal toxicities (9, 22, 23). At this stage, serum phosphate should be regarded as a candidate metabolic correlate that may inform future research, rather than a therapeutic target.

### Strengths and limitations

The major strengths of this study include its large sample size (N = 2, 226), which provided 90% statistical power to detect the primary association; the use of live birth as a clinically meaningful primary outcome; the detailed analysis of the embryological cascade; and the adjustment for trigger-day progesterone to control for premature luteinization in fresh cycles. The measurement-window analysis provided biologically relevant temporal context, and sensitivity analyses accounted for calcium, alkaline phosphatase, fasting glucose, and trigger-day progesterone.

Several limitations must be acknowledged. First, the retrospective single-center design limits causal inference and generalizability. Second, the study was restricted to fresh embryo transfer cycles to reduce heterogeneity related to freeze-thaw procedures, endometrial preparation protocols, transfer timing, and variable storage intervals. However, frozen embryos originate from the same oocyte cohort and follicular environment; therefore, exclusion of frozen cycles may introduce selection bias and should be addressed in future studies. Third, important regulators of phosphate homeostasis, including PTH, 25-hydroxyvitamin D, FGF23, klotho, and dietary phosphate intake, were unavailable, precluding dissection of the underlying metabolic pathways. Fourth, follicular fluid phosphate was not measured. Paired follicular fluid and serum measurements are needed in future studies to directly evaluate the oocyte microenvironment. Fifth, detailed embryo kinetic parameters, ploidy status, semen parameters, and sperm DNA fragmentation were unavailable. Sixth, residual confounding by lifestyle, nutrition, and unmeasured reproductive factors cannot be excluded. Finally, no correction for multiple comparisons was applied to exploratory subgroup analyses.

## Conclusions

Higher pre-treatment fasting serum phosphate was associated with higher odds of live birth after IVF/ICSI fresh embryo transfer, independent of MII oocyte count and usable embryo count. This association was temporally consistent across the pre-treatment period, particularly within the 60–120 day window. Serum phosphate represents a novel, inexpensive metabolic correlate of reproductive competence. External validation in prospective cohorts, including frozen embryo transfer cycles and serial measurements of phosphate-regulating hormones, is required before clinical application.

## Data Availability

The original contributions presented in the study are included in the article/**Supplementary Material**. Further inquiries can be directed to the corresponding authors.
