# Supplementary material for: Pre-treatment serum phosphate as a metabolic correlate of live birth after IVF/ICSI: a retrospective cohort study
Source: Front Endocrinol (Lausanne). 2026 Aug 27;17:1938890. doi: 10.3389/fendo.2026.1938890 (PMC13557955; doi:10.3389/fendo.2026.1938890)
Supplement: Supplementary file 1 [file Table1.docx]

Table S1. Sensitivity analyses adjusting for trigger-day progesterone and metabolic markers linked to phosphate homeostasis

Outcome: live birth. Exposure: serum phosphate per 1-SD increase (0.146 mmol/L)

| Models | N | OR (95% CI) | P value |
| --- | --- | --- | --- |
| Model 3 (Primary) | 2226 | 1.15 (1.06-1.26) | 0.002 |
| Model 3 + trigger-day progesterone | 2226 | 1.15 (1.05 to 1.26) | 0.002 |
| Model 3 + Calcium | 2226 | 1.16 (1.06-1.27) | 0.001 |
| Model 3 + ALP | 2225 | 1.15 (1.06-1.26) | 0.002 |
| Model 3 + Fasting Glucose | 2226 | 1.16 (1.06-1.27) | 0.001 |
| Model 3 + Ca + ALP + Glucose | 2225 | 1.16 (1.06-1.27) | 0.001 |

Models assess whether the phosphate-live birth association is attenuated after adjustment for trigger day progesterone, calcium (proxy for PTH activity), alkaline phosphatase (bone turnover/vitamin D marker), and fasting glucose (insulin-mediated phosphate uptake). OR, odds ratio; CI, confidence interval; ALP, alkaline phosphatase.

Table S2. Oocyte and embryological indicators of the study cohort stratified by pre-treatment serum phosphate quartile (N = 2,226).

|  | Serum Phosphorus Quartiles | | | |
| --- | --- | --- | --- | --- |
|  | Q1 N = 591 | Q2 N = 558 | Q3 N = 542 | Q4  N = 535 |
| Total oocytes retrieved | 5791 | 5532 | 5274 | 5337 |
| Total MII oocytes | 5107 | 4925 | 4689 | 4751 |
| Total 2PN embryos | 4316 | 4189 | 3985 | 4035 |
| Total usable embryos | 2612 | 2506 | 2343 | 2400 |
| Oocyte maturation index | 0.88 | 0.89 | 0.89 | 0.89 |
| Aggregate fertilization rate | 0.85 | 0.85 | 0.85 | 0.85 |
| Aggregate embryo utilization rate | 0.61 | 0.60 | 0.59 | 0.59 |
| Women with ≥1 cryopreserved embryo, n (%) | 512/591 (86.6) | 484/558 (86.7) | 467/542 (86.2) | 470/535 (87.9) |

Oocyte maturation index was calculated as total MII oocytes divided by total oocytes retrieved. Aggregate fertilization rate was calculated as total 2PN embryos divided by total MII oocytes. Aggregate embryo utilization rate was calculated as total usable embryos divided by total 2PN embryos, according to the Vienna consensus on ART laboratory performance indicators.
